# Glucocorticoid induced adrenal insufficiency is common in steroid treated glomerular diseases - proposed strategy for screening and management

**DOI:** 10.1186/s12882-019-1354-6

**Published:** 2019-05-06

**Authors:** Alvin H. K. Karangizi, May Al-Shaghana, Sarah Logan, Sherwin Criseno, Rachel Webster, Kristien Boelaert, Peter Hewins, Lorraine Harper

**Affiliations:** 1 Institute of Clinical Sciences, College of Medical and Dental Sciences, University of Birmingham, Birmingham, B15 2TT UK; 2 Department of Renal Medicine, Queen Elizabeth Hospital Birmingham, University Hospitals Birmingham NHS Trust, Birmingham, B15 2TH UK; 3 Department of Endocrinology, Queen Elizabeth Hospital Birmingham, University Hospitals Birmingham NHS Trust, Birmingham, B15 2TH UK; 4 Department of Biochemistry, Queen Elizabeth Hospital Birmingham, University Hospitals Birmingham NHS Trust, Birmingham, B15 2TH UK; 5 Institute of Metabolism and Systems Research, College of Medical and Dental Sciences, University of Birmingham, Birmingham, B15 2TT UK

**Keywords:** Steroids, Medication, Screening, Renal disease, Cortisol, Short synacthen test, Adrenal insufficiency

## Abstract

**Background:**

Glucocorticoids (GCs) are frequently used to treat glomerular diseases but are associated with multiple adverse effects including hypothalamic-pituitary-adrenal axis inhibition that can lead to adrenal insufficiency (AI) on withdrawal. There is no agreed GC tapering strategy to minimise this risk.

**Methods:**

This is a single centre retrospective study, between 2013 to 2016, of patients with glomerular disease on GC therapy for more than 3 months screened for GC induced AI with short synacthen stimulation tests (SSTs) done prior to complete GC withdrawal. We investigated the prevalence of AI, predictors, choice of screening tool and recovery.

**Results:**

Biochemical evidence of GC induced AI was found in 57 (46.3%) patients. Total duration of GC did not differ between those with and without AI (*p* = 0.711). Patients with GC induced AI had a significantly lower pre-synacthen baseline cortisol as compared to patients without AI. A cut off pre-synacthen baseline cortisol of ≥223.5 nmol/l had a specificity of 100% for identifying individuals without biochemical AI. Patients with GC induced AI took a mean of 8.7 ± 4.6 months (mean ± SD) to recover. Patients with persistent AI had a significantly lower index post-synacthen cortisol measurement.

**Conclusions:**

We demonstrate that biochemically proven GC induced AI is common in patients with glomerular diseases, is not predicted by daily dose or duration and takes a considerable time to recover. The study supports the use of morning basal cortisol testing as an appropriate means to avoid the need for SSTs in all patients and should be performed in all patients prior to consideration of GC withdrawal after 3 months duration.

## Background

Glucocorticoids (GCs) are a widely used and effective treatment for glomerular diseases such as lupus nephritis, ANCA associated vasculitis and primary glomerular diseases. GCs are effective in managing these conditions but are associated with significant adverse effects such as weight gain, diabetes mellitus, stomach ulceration, gastrointestinal bleeding, infection and osteoporosis. To minimise these risks, maintenance doses are generally kept as low as possible and cessation is considered in patients in stable remission [1].

GCs inhibit the hypothalamic-pituitary-adrenal (HPA) axis through negative feedback. Chronic inhibition can result in adrenal insufficiency (AI) which is unmasked on withdrawal. This can be serious and potentially life threatening as patients are unable to mount an adequate cortisol stress response. The prevalence of AI after oral administration of corticosteroids is common with estimates ranging from 14 to 63% [2]. The dose and duration related threshold for developing AI is unclear. Studies show a relationship between dose and duration of therapy, however this has not been replicated in all studies and AI may develop in those perceived to be at low risk with total cumulative doses of Prednisolone less than 500 mg and duration of therapy less than 4 weeks [2]. There are no evidence-based guidelines to support current practice on tapering of GCs [1]. Clinical practice in most centres is to gradually taper the dose of prednisolone with the expectation that this will enable adrenal recovery. To the contrary, a recent systematic review showed that adrenal suppression can persist after withdrawal with an absolute risk of 25.3% (CI 19.4 to 32.3%) at 6 months post cessation [3].

Existing literature is conflicting regarding strategies to mitigate this risk with some suggesting the need fora low index of suspicion to test patients following withdrawal and others advising switching to hydrocortisone during tapering [1, 3]. In order to minimise the risk of AI following steroid withdrawal, we adopted the approach of screening patients with a short synacthen stimulation test (SST) prior to GC withdrawal. We have now audited this practice to investigate its value in terms of, identifying prevalence of biochemical AI, recovery and screening tool choice. To the best of our knowledge, no previous studies have specifically investigated GC induced AI in glomerular disease.

## Methods

### Study design, settings and participants

This was a single-centre retrospective observational service evaluation carried out in the Renal Department at the Queen Elizabeth Hospital, Birmingham, UK. Patients were identified using data log of all SSTs carried out by our Kidney Assessment Team over a 3 year time period, between September 2013 and September 2016. We included all patients taking Prednsiolone, for longer than 3 months, for glomerular disease who were screened for AI prior to intended complete withdrawal from GCs. We excluded patients on GCs for indications other than glomerular disease, and patients who had SSTs performed for indications other than planned GC withdrawal This study received approval from the clinical audit department at the Queen Elizabeth Hospital Birmingham, UK in 2014 (Audit Code: CARMS-11451).

We collected data on patient age, sex, renal diagnosis, date of diagnosis, date when GC commenced, highest recorded dose of Prednsiolone, use or non-use of Methylprednisolone on induction, Prednsiolone dose at time of SST, duration of Prednsiolone use, concurrent inhaled or topical GC use, SST result, time to retest and time to and result of subsequent SSTs where necessary. Data was collected from our electronic medical record system of test results and clinic letters.

### Adrenal insufficiency definition and assay

Adrenal function was assessed by a Short Synacthen stimulation Test (SST) performed between 08:30 am and 10:00 am after an overnight fast and 24 h Prednisolone pause. Cortisol levels were measured before and 30 min after a bolus of 250 μg tetracosactide (Synacthen, *Ciba-Geigy*) administered intramuscularly. Tetracosactide is a synthesized polypeptide with an amino acid sequence including 1 to 24 of the 1 to 39 chain of the naturally occurring corticotropin. It has adrenocortical stimulatory action similar to that of natural coticotropin [4]. It has previously been established that the 30-min cortisol level is used as the criterion to define adequate or inadequate adrenal cortisol reserve, and is the standard by which decisions are made to instigate (or terminate) glucocorticoid replacement [5]. The 30-min cortisol level provides reliable measure of adrenal function on the day of the test and whether this is adequate or not [5–7]. All synacthen tests were performed in the University Hospitals Birmingham NHS foundation Trust by trained nurses and the samples were analysed in the Biochemistry Laboratories. During the initial data collection period, our laboratory used the *Roche cobas e602 Gen 1 competitive immunoassay* which had a 30 min pass SST cut-off of cortisol ≥550 nmol/l derived using an in-house study of 100 healthy subjects. In January 2016 there was a switch to a new assay, *Roche cobas e602 Gen 2 competitive immunoassay*, due to restandardisation with cortisol mass spectrometry methodology and data quality assurance schemes to align more closely to reference material. The Gen 2 assay was shown to have a − 18.25% negative bias and the 30 min SST pass cut-off was changed to ≥450 nmol/l to reflect this. Following the change, an internal audit was performed to assess the number of failed SSTs following the assay change. There was no significant difference before or after. As of mid-2017 (because of a shortage of Synacthen supply), the endocrine department now recommends a morning 9 am basal cortisol as the baseline screening tool for adrenal insufficiency with an adequate cortisol defined as ≥350 nmol/l. Patients with a cortisol measurement of < 350 nmol/l require a SST to confirm adrenal function. Basal ACTH was not measured in this study.

### Follow-up process

Patients who failed the SST were diagnosed with biochemical AI and referred for review by an endocrine specialist nurse. They were switched to hydrocortisone, a short-acting synthetic glucocorticoid dosed at 10 mg in the morning and 5 mg in the evening to try to mimic endogenous cortisol production. Patients were educated on sick day rules including the need to double their doses in times of illness. They were then retested at 6 months, with hydrocortisone withheld on the morning of the synacthen test. Patients were retested at 6 monthly intervals until recovery.

### Statistical analysis

Data are presented as mean ± standard deviation for parametric variables and median (interquartile range) for non-parametric variables. Categorical variables have been expressed as counts (percentage). Between groups comparisons were performed using Mann Whitney U test, t-test or Chi squared test as appropriate. A receiver-operating characteristic (ROC) curve was performed to compute the sensitivity and specificity of the pre-synacthen baseline cortisol in predicting the outcome of the SST test. Statistical analysis has been performed using GraphPad Prism Version 5.00 (for Windows, 2007) and SPSS version 22 (IBM Corp, 2013).

## Results

### Patient characteristics

One hundred and twenty three patients were included in the study; the characteristics of the patients at the time of SST are shown in Table 1. Primary glomerular diseases represented in the study included IgA nephropathy, IgA vasculitis, minimal change disease, idiopathic membranous nephropathy, focal segmental glomerulosclerosis and C3d glomerulonephritis. At the time of SST testing, all patients were on Prednisolone doses of 5 mg or less. 80 (65%) of the patients had been on a dose of 5 mg or less for at least 6 months before the test with the remaining patients receiving higher doses during this period. The duration of Prednisolone use was available for 63 patients (51%) and ranged from 4 to 100 months (Table 1). Data on use or non-use of intravenous Methylprednisolone at induction was available for 93 patients (75%) (Table 1). The initial daily dose of Prednisolone was available for 79 patients (64%) (Table 1). 7 patients were concurrently using GC inhalers. There was no documented concurrent use of topical GCs.

**Table 1:** Baseline characteristics of all patients

Variable		Result (*n* = 123)
Age (years)		51.9 years ±18.27
Sex (count)	Male	63 (51.2%)
	Female	60 (48.8%)
Renal diagnosis (count)	ANCA-associated vasculitis	50 (35.7%)
	Primary Glomerular disease	33 (26.8%)
	Lupus nephritis	30 (24.4%)
	Other	10 (13.0%)
Renal function (eGFR by MDRD^a^ equation)		66 (39–90)
Duration of Prednisolone use (months) (data available for *n* = 63)		18 (9–35)
Induction with intravenous methylprednisolone (count) (data available for *n* = 93)		19 (20%)
Initial daily Prednisolone dose (mg) (data available for *n* = 79)		50 (30–60)
Concurrent GC inhaler use (count)		7 (5.7%)

^a^eGFR by MDRD – Estimated Glomerular Filtration Rate by Modification of Diet in Renal Disease Equation

### Adrenal function

**Fig. 1:**
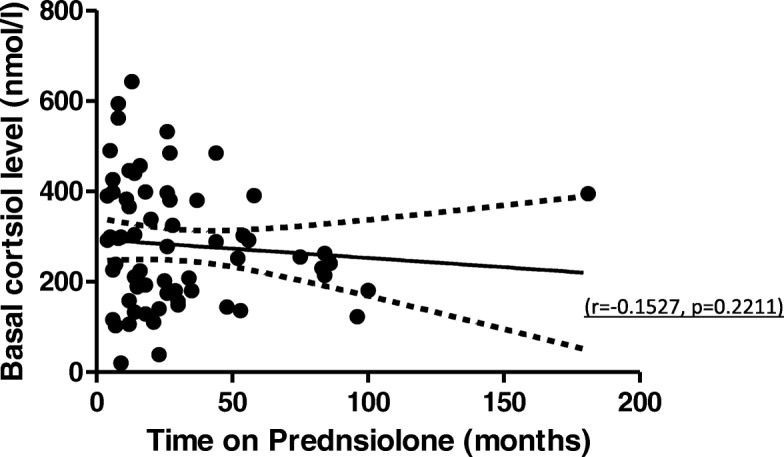
Scatter graph of duration of prednisolone use plotted against pre-synacthen baseline cortisol result

57 (46.3%) patients had an insufficient adrenal response to the SST and were therefore diagnosed with biochemical AI. There were no documented clinical episodes of AI prior to the initial SST. The mean baseline cortisol of patients who passed the SST was significantly higher than those who failed with both assays (Table 2). There was no significant difference in age, sex, total duration of Prednisolone use, use of intravenous Methylprednisolone at induction, initial Prednisolone dose, concurrent GC inhaler use and degree of renal impairment between the adrenal sufficient and insufficient patients (Table 2). The outcome was also not influenced by whether or not the patient had been on Prednisolone of 5 mg or less for at least 6 months before the test. There was no significant correlation between duration of Prednisolone and pre-synacthen baseline cortisol (Fig. 1). Underlying glomerular disease did not influence SST outcome (*p* = 0.193).

**Table 2:** Differences between patients who pass and fail SST

Variable	SST Pass	SST Fail	*P* value
Age (years)	53 ± 2.3	51 ± 2.4	0.664
Duration of Prednisolone use (months)	17 (8–44)	22 (12.3–34.5)	0.711
Induction with intravenous Methylprednisolone (count)	11 (22%)	8 (19%)	0.686
Initial daily Prednisolone dose (milligrams)	60 (30–60)	40 (30–60)	0.315
GC inhaler use (count)	2 (3.0%)	5 (8.9%)	0.163
Prednisolone 5 mg or less for at least 6 months (count)	42 (64%)	38 (69%)	0.528
Renal function (eGFR by MDRD^1^ equation)	69 (40–90)	64 (37–89)	0.294
Pre synacthen baseline Cortisol (1st assay) (nmol/l)	380 ± 19 (*n* = 54)	200 ± 15 (*n* = 38)	< 0.001
Pre synacthen baseline Cortisol (2nd assay) (nmol/l)	269 ± 31 (*n* = 11)	133 ± 20 (*n* = 18)	< 0.001

### Switch to morning 9 am cortisol test

**Fig. 2:**
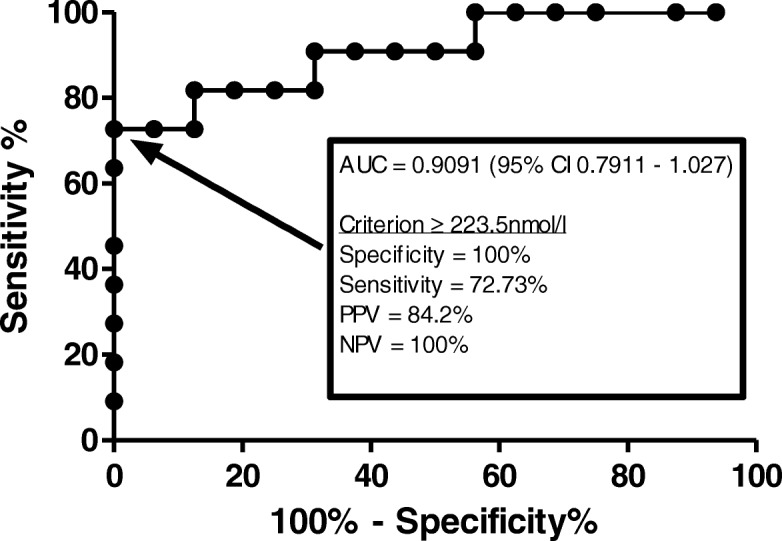
Pre-synacthen cortisol level as a predictor of SST outcome. Receiver-operating characteristic curve with true positive rate (sensitivity %) plotted against false positive rate (100% - specificity%) for different cut-off levels

A ROC curve was performed using results of SSTs performed with the Gen 2 assay (*n* = 27) (Fig. 2). A pre-synacthen baseline cortisol (taken between 8:30 am and 10:00) ≥223.5 nmol/l had a specificity of 100% for passing the SST and a sensitivity of 72.73%. Values above this threshold had a negative predictive value (baseline result above this level indicates healthy individual) of 100% and a positive predictive value (baseline result below this level indicates AI) of 84.2%. A pre-synacthen baseline cortisol ≤119.5 nmol/l had a sensitivity of 100% for identifying patients with biochemical AI. Utilising this lower cut-off and the assay validated ≥350 nmol/l upper cut-off would have resulted in 33.3% (*n* = 9) SSTs being avoided.

### Follow-up

Thirty-eight (66.6%) of the 57 patients that failed the SST have had follow-up re-testing. Of the 18 patients that have not been re-tested, 4 are currently waiting for the test, 1 patient died, 8 patients had disease relapse and clinical indication to continue Prednisolone, 3 patients have opted to continue Prednisolone and 3 patients have been lost to follow-up. Of the 38 patients that have been re-tested, 1 patient opted to go back on Prednisolone and a further 4 had disease relapse and indication to continue Prednisolone. Of the remaining 33 patients, 18 patients have recovered adrenal function with a mean time of recovery of 8.7 ± 4.6 months and 15 patients have not recovered with latest testing at a mean of 15.9 ± 9.2 months. Patients who have recovered from AI had a significantly higher post synacthen cortisol concentration at their initial SST screen than patients who have not recovered across both assays (Table 3). There was no significant difference in age, duration of prednisolone use, initial prednisolone dose and initial pre synacthen baseline cortisol between the two groups. The latest recovery was 23 months following discontinuation of prednisolone.

**Table 3:** Differences between patients who have recovered from AI and those who have not

Variable	Recovered (*n* = 18)	Not recovered (*n* = 15)	*P* Value
Age (years)	53 ± 3.4	48 ± 4.8	0.451
Duration of Prednisolone use (months)	28 ± 25.7	25 ± 16	0.888
Initial Prednisolone dose (milligrams)	40 (IQR 30 to 60)	30 (IQR 25 to 55)	0.367
Index screen pre synacthen baseline cortisol 1st assay (nmol/l)	205 ± 30 (*n* = 14)	202 ± 31 (*n* = 9)	0.962
Index screen pre synacthen baseline cortisol 2nd assay (nmol/l)	208 ± 8.3 (*n* = 3)	134 ± 30 (*n* = 6)	0.148
Index screen post synacthen cortisol 1st assay (nmol/l)	439 ± 25 (*n* = 14)	343 ± 37 (*n* = 9)	0.034
Index screen post synacthen cortisol 2nd assay (nmol/l)	394 ± 24 (*n* = 3)	240 ± 38 (*n* = 6)	0.033

## Discussion

This study has demonstrated that GC induced AI is common (46.3%) in patients with glomerular disease and is present prior to complete withdrawal of Prednisolone. To the best of our knowledge, this has not previously been shown in a subgroup of patients on GCs for glomerular disease. Our results reflect similar prevalence of AI to patients with rheumatoid arthritis receiving low dose GCs [3]. Further analysis of this cohort has shown that age, gender, underlying renal disease, duration of Prednisolone use, use of intravenous Methylprednisolone at induction, starting Prednisolone dose, concurrent GC inhaler use and degree of renal impairment do not influence the risk of AI. We have also shown that keeping patients on maintenance Prednisolone doses of 5 mg or less for at least 6 months does not lessen the risk. The literature is conflicting over the effect of renal impairment on the HPA axis [8, 9]. One study, with a limited number of subjects, found comparable responses to ACTH between patients with renal impairment and controls [10]. These results reinforce the need for a low index of suspicion when withdrawing GCs, even in patients on low doses.

Our study adds to the current understanding of AI by identifying thresholds of baseline cortisol levels for undertaking SSTs. Random cortisol measurements are not a reliable assessment of the HPA axis due to the diurnal rhythm variation of secretion. Production however peaks at around 08:00 to 09:00 am and previous studies have shown a strong correlation between the basal morning serum cortisol (08:00 to 09:00 am) and the peak cortisol level after ACTH administration [11]. The mean pre-synacthen baseline cortisol, taken between 8:30 am and 10:00 am, in this study was significantly lower in patients who failed the SST. ROC analysis showed that a pre-synacthen basal cortisol ≥223.5 nmol/l had a specificity of 100% for identifying individuals with preserved adrenal function and therefore supports the use of a morning 9 am basal cortisol as an initial screen for AI (validated cut off ≥350 nmol/l). In addition we found that a basal cortisol < 119.5 mol/l adequately identified patients with biochemical AI. We found that utilising upper and lower limit cut offs would have avoided the need for a SST in a third of patients. Indeed a previous study found that the SST adds little value for a basal cortisol of less than 100 nmol/l or more than 500 nmol/l [12]. A worldwide shortage of Synatchen in 2014 led to a substantial increase in price and avoidance of use may provide a financial benefit [13].

A strength of our study is that we ensured that patients were off Prednisolone for 24 h prior to assessment of adrenal function. All common corticosteroids except Dexamethasone can cross-react with the assay to some degree. The half-life for these agents is approximately 0.5–4.0 h so it is important that Prednisolone is paused for at least 24 h prior to baseline cortisol measurements [14, 15]. We switched patients identified with AI to twice daily Hydrocortisone which has a shorter half-life than Prednisolone as well as being more favourable on bone mineral density [16]. Patients are also educated on sick day rules and retested at 6 monthly intervals until recovery.

Our study also adds to current understanding by evaluating duration and predictors of recovery. We have shown that patients can take a considerable time to recover which raises concerns over the safety of conventional practice of unmonitored tapering. A higher initial post synacthen cortisol concentration can predict earlier recovery and therefore could inform prognosis discussions with patients. The latest recovery was 23 months after discontinuation of Prednisolone with not a single patient recovering more than 2 years following Prednisolone discontinuation (three patients with latest follow-up at 27, 33 and 36 months respectively). This finding suggests that there may be a cohort of patients that do not recover and have permanent AI. A similar study done in patients with inflammatory bowel disease found a longer time to recovery with a mean of 3.1 ± 2.5 years (mean ± SD) however comparison between this study and our findings is limited by the small sample sizes in both cohorts. The longer time to recovery found in this study does however demonstrate delayed recovery and supports the need to continue re-testing patients with biochemical adrenal insufficiency even after 2 years of discontinuation [17].

One of the limitations of this study was its retrospective design, which led to incomplete access to data on duration of Prednisolone and initial dose, including use of intravenous glucocorticoids on induction. This design may have also led to inaccurate figures on concurrent use of alternate forms of GCs and selection bias due to inability to capture data on patients lost to follow-up. Patients were not instructed to withhold inhaled steroid preparations on the day of the synacthen test which may have suppressed the morning cortisol level, however, from the available data; only 7 patients were using concurrent steroid inhalers. We found no association between duration of GC use and AI, however it must be noted that all our patients had received at least 4 months of GC therapy. The risk of AI has been noted to increase with duration of therapy in many studies but there is considerable variation reported. The relatively small sample sizes in this study may have led to it being underpowered to investigate the impact of initial daily dose and duration of therapy. SST testing, although convenient, is not as accurate as testing the entire HPA axis with alternate tests such as the overnight metyrapone test or insulin tolerance test [18]. We used the standard dose SST (250 μg synacthen) rather than the low dose SST (1 μg synacthen). The later has shown a high intermethod variability in dilution strategies which may result in false positive rates impacting on test specificity [19]. There was a switch in assay towards the end of the data collection window, which consequently resulted in smaller sample sizes when analysing for differences in cortisol levels and outcomes separately for each of the assays. Despite this we still found significant relationships between a lower baseline cortisol in patients who failed the SST and a higher post synacthen cortisol in the recovery group across both assays. An in house audit found no significant difference in number of failed SSTs following the assay change and it is therefore unlikely that the change in assay had an effect on the results.

## Conclusions

In summary, we have demonstrated that biochemical GC induced AI is common in steroid treated glomerular diseases. Initial Prednisolone dose and duration of use did not predict the risk. 9 am basal cortisol is an appropriate first line screening tool and avoids the need for a SST in some patients. Our proposed approach of screening patients, hydrocortisone replacement and education aims to protect patients at risk of adrenal crisis.

## Abbreviations

ACTH: Adrenocorticotropic hormone
AI: Adrenal Insufficiency
ANCA: Antineutrophil cytoplasmic antibodies
eGFR: estimated Glomerular Filtration Rate
GC: Glucocorticoids
GN: Glomerulonephritis
HPA: Hypothalamic-pituitary-adrenal axis
MDRD: Modification of Diet in Renal Disease equation
ROC: Receiver Operating Curve
SST: Short Synacthen Test
